# Sleep problems in adolescents with CFS: A case-control study nested within a prospective clinical cohort

**DOI:** 10.1177/1359104520918364

**Published:** 2020-05-22

**Authors:** Maria Elizabeth Loades, Katharine A Rimes, Trudie Chalder

**Affiliations:** 1 Department of Psychology, University of Bath, UK; 2 Bristol Medical School, University of Bristol, UK; 3 Department of Psychological Medicine, Institute of Psychiatry, Psychology and Neuroscience, King’s College London, UK; 4 South London & Maudsley NHS Foundation Trust, UK

**Keywords:** Adolescents, chronic fatigue syndrome, fatigue, insomnia, sleep

## Abstract

Sleep problems have a negative impact on a range of outcomes and are very common in adolescents with chronic fatigue syndrome (CFS). We aimed to (a) establish whether adolescents with CFS have more self-reported sleep problems than illness controls as well as healthy controls, (b) investigate changes in sleep problems and (c) explore the extent to which sleep problems at baseline predict fatigue and functioning at follow-up in adolescents with CFS. The Insomnia Scale was completed by 121 adolescents with CFS, 78 healthy adolescents and 27 adolescents with asthma. Eighty (66%) treatment-naïve adolescents with CFS completed questionnaires approximately 3 months later. Adolescents with CFS reported increased sleep problems compared to healthy controls and adolescents with asthma. In CFS, there was no significant change in sleep problems without treatment over a 3-month follow-up. Sleep problems at baseline predicted a significant proportion of the variance in sleep problems at follow-up. Sleep problems should be targeted in treatment. Regulating the ‘body clock’ via the regulation of sleep could influence outcomes not assessed in this study such as school attainment.

## Background

Chronic fatigue syndrome (CFS) is characterised by ongoing severe and disabling fatigue, of at least 3 months duration, which is not the result of another medical condition (NICE, 2007). Other accompanying dificulties can include sleep disturbances, as well as attention and concentration problems, nausea, dizziness, headaches and muscle and joint pain (NICE, 2007). It affects approximately 1–2% of adolescents (Brigden et al., 2017) and causes significant disability, including significantly decreased school attendance (Crawley & Sterne, 2009).

Sleep problems are common in adolescents with chronic health conditions and may result from underlying disease-related pathology, treatment regimens or inpatient hospital stays (Lewandowski et al., 2011). In adolescents with chronic pain, for example, sleep problems are common and persistent and predict poorer quality of life over time (Palermo et al., 2012). Furthermore, lower subjective sleep quality is associated with higher activity limitations, assessed objectively using actigraphy (Palermo et al., 2008).

Insomnia is a term generally used to describe difficulties falling asleep or staying asleep and is more specifically defined in diagnostic manuals as difficulty initiating or maintaining sleep, or having non-restorative sleep despite having adequate opportunity for sleep that interferes significantly with functioning (American Psychiatric Association, 2013). Despite sleep disturbances being common in chronic health conditions and being specifically included as a possible concomitant symptom in the diagnostic criteria for CFS, there has been little research attention given to sleep in adolescents with CFS.

The normal 24-hour rhythms of sleep and wakefulness are maintained by a body clock mechanism, which utilises information about the external world from photoreceptors within the eyes as well as internal information about sleep pressure which builds up during wakefulness, to regulate sleep (Foster & Kreitzman, 2014). Daily routines such as mealtimes and regular activities like getting dressed and being engaged in activity during the day also contribute to the rhythm (Yoshizaki et al., 2013). Obtaining the optimal amount of sleep has been linked to improved concentration and attention (Perez-Lloret et al., 2013) and better functioning across a range of outcomes (Walker, 2017). As part of normative development, sleep patterns change during adolescence. Normative changes include a reduction in total sleep duration, a shift towards later bedtimes, longer time to fall asleep (sleep onset latency) and increased variability between sleep patterns during the week and at weekends (Carskadon et al., 1993; Crowley et al., 2007; Gradisar et al., 2011). Behavioural factors such as screen usage have been found to be associated with shortened sleep duration and delayed sleep onset (Hale & Guan, 2015; Hysing et al., 2015), and this sleep disturbance may contribute to developing symptoms of depression (Lemola et al., 2015). Cognitive factors such as catastrophising have also been associated with sleep disturbance in adolescents (Gregory et al., 2010; Hiller et al., 2014). Adolescents with CFS specifically endorse more catastrophic thinking about symptoms than those with asthma (Loades, Rimes, Lievesley, et al., 2019). Sleep difficulties are known to be associated with depression and anxiety (Alfano et al., 2007, 2009; Lovato & Gradisar, 2014; Lovato et al., 2017), and the prevalence of depression and anxiety is particularly high in adolescents with CFS (Loades et al., 2018).

Beyond normative changes in sleep seen in the general adolescent population, sleep disturbance is a very common concomitant symptom in adolescents with CFS, reported in more than 95% of 12- to 18-year-old patients (Collin et al., 2015), with difficulties waking up and feeling unrefreshed highlighted as a key feature of CFS by affected adolescents (Parslow et al., 2018). It may be that adolescents with CFS sleep for longer in the hope that sleep will be restorative and that they will wake up feeling less fatigued. A large-scale cohort study found that adolescents who had chronic disabling fatigue were more likely to have had a shorter sleep duration and more difficulties going to sleep in childhood, which suggests that sleep abnormalities may have a causal role in CFS, or share a pathophysiological cause (Collin et al., 2018). Similar to findings in the otherwise healthy adolescent population, poor sleep can impact adversely on educational, cognitive, physical and mental health and social outcomes in paediatric CFS (Josev et al., 2017).

Despite its common occurrence, there is a relative dearth of empirical research on sleep in paediatric CFS. A review found only six studies of sleep in paediatric CFS, five case-control studies and one case series (Snodgrass et al., 2015). Adolescents with CFS tended to experience more difficulties with sleep than healthy controls, but conclusions drawn are limited by small sample sizes, varying diagnostic criteria for CFS and the lack of an illness control group. Studies published since this review have yet to comprehensively address these issues. Josev et al. (2017) found that adolescents with clinician confirmed CFS (*N* = 21) had a significantly longer objective sleep onset latency, spent more time in bed, had a greater total sleep time and later rise time and had significantly poorer subjective sleep quality compared to healthy controls. Higher anxiety was associated with poorer subjective sleep quality in both groups. Pedersen et al. (2017) also found that adolescents with clinician confirmed CFS (*N* = 120) stayed in bed for longer, had significantly more varied delayed sleep rhythms and reported more insomnia symptoms including sleepiness, problems waking up and taking longer to fall asleep than healthy controls. Although both studies included objective and subjective measures of sleep, neither study included an illness control group and neither study followed up the participants over time to examine how sleep difficulties affected prognosis. Thus, it is yet to be established whether the differences in sleep, between those with CFS and illness controls, are specific to CFS, or may be associated with illness more broadly.

Adolescents with CFS experience disturbed sleep compared to healthy adolescents, but little is known about whether these disturbances are specific to CFS, or related to prognosis. The current study aimed to examine self-reported sleep disturbances in a clinical cohort of adolescents with CFS compared to adolescents with asthma (illness controls) and healthy adolescents and to investigate the impact of sleep disturbances on outcome in CFS. Asthma is a chronic illness with an unpredictable and variable nature, requiring ongoing medical monitoring but is usually well controlled.

The hypotheses were as follows:

1. Participants with CFS will report greater sleep problems than adolescents with asthma and healthy adolescents.
2. In participants with CFS, elevated sleep problems at baseline will be ongoing at follow-up.
3. Elevated sleep problems at baseline will explain some of the variance in the outcomes of fatigue and functioning at follow-up in treatment-naive adolescents with CFS, even when controlling for other covariates (mood, anxiety, catastrophising, age as a proxy for pubertal status and time elapsed between baseline and follow-up).

## Method

### Participants

We recruited three groups of adolescents (age = 11–18 years). The CFS group were required to have a clinician confirmed diagnosis of CFS (NICE, 2007), which, by definition, excludes those with a primary mental health problem. Between August 2010 and January 2012, all eligible adolescent patients who attended an initial clinical assessment at two specialist CFS units in London were invited to participate. Recruitment continued at one unit until October 2017. During the recruitment period, a total of 207 adolescents attended an assessment. Of these, 135 met the eligibility criteria and 121 (89.6%) participated (see Table 1 for participant demographics).

**Table 1.** Participant demographics.

	CFS participants (*n* = 121)	Asthma participants (*n* = 27)	Healthy controls (*n* = 78)
Age (mean)	15.0	14.9	14.6
Gender – *N* (%)			
Males	35 (28.9)	12 (44.4)	30 (38.5)
Females	86 (71.1)	15 (55.6)	48 (61.5)
Ethnic background – *N* (%)			
White British	86 (71.1)	16 (59.3)	65 (83.3)
Black British	2 (1.7)	1 (3.7)	1 (1.3)
Asian/British Asian	3 (2.5)	2 (7.4)	2 (2.6)
Other British/European/White	25 (20.7)	7 (25.9)	
Other Black/Asian			4 (5.2)
Mixed race	4 (3.3)		2 (2.6)
Not stated	4 (3.3)	1 (3.7)	4 (5.1)

CFS: chronic fatigue syndrome.

Asthma participants were required to be prescribed medication for asthma and have no known history of mental health problems. Healthy participants had no history of CFS, asthma or mental health problems.

### Measures

All participants completed the measures on one occasion at time 1. Those with CFS who attended for follow-up completed measures again at 3 months prior to any treatment being given.

#### Sleep

The Insomnia Scale (Abdel-Khalek, 2008) is a 12-item self-report scale which assesses difficulties with sleeping. Each item is answered on a 5-point scale (0–4). This scale is composed of two factions: difficulty in initiating sleep and maintaining sleep (sleep problems) and consequences of sleep. It is scored as one total score, which is the sum of the responses to each of the 12 items. Higher scores indicate greater sleep problems. The Insomnia Scale has acceptable test–retest alpha reliabilities and good convergent validity including in adolescent populations (Abdel-Khalek, 2008). In this study, Cronbach’s alpha was .86 (CFS participants), .92 (asthma participants) and .90 (healthy controls).

#### Fatigue

The Chalder Fatigue Questionnaire (CFQ; Chalder et al., 1993) contains 11 items to measure severity of fatigue (both mental and physical) over the past month. Each item is rated on a 4-point scale (0–3). We used the Likert-type method of scoring, resulting in a possible maximum score of 33. Higher scores indicate greater fatigue severity. The CFQ has established reliability and validity (Cella & Chalder, 2010; Chalder et al., 1993) and it has been extensively used in studies of adolescents with CFS (Baos et al., 2018; Brigden et al., 2016; Lloyd, Chalder, & Rimes, 2012; Lloyd, Chalder, Sallis, & Rimes, 2012). Cronbach’s alpha was .89 (CFS participants), .66 (asthma participants) and .82 (healthy controls).

#### Physical functioning

The Short Form 36 Physical Functioning Scale (SF-36PFS; Ware & Sherbourne, 1992) has 10 items. It assesses how limited a respondent is by their health problems across various activities of daily living. Each item is rated on a 3-point scale (scored 0, 5 or 10), with a possible maximum score of 100, calculated by summing the scores on each item. Higher scores indicate better physical functioning. The SF-36 has been previously validated in adolescent chronic illness samples, for example, cystic fibrosis (Gee et al., 2002). Cronbach’s alpha was .91 (CFS participants), .72 (asthma participants) and .90 (healthy controls).

#### Anxiety

The State-Trait Anxiety Inventory (STAI; Spielberger et al., 1970) is a 40-item measure; 20 items assess general threat sensitivity (‘trait anxiety’) and 20 assess anxiety in response to particular threats (‘state anxiety’). Each item is rated on a 4-point scale (scored 1–4). A number of items are reverse coded. Subscale scores are calculated by summing the scores for the relevant items. Higher scores indicate greater anxiety. Valid and reliability have been established previously (Spielberger et al., 1970), including in paediatric samples (Spielberger, 1983). Cronbach’s alpha for the state items was .93 (CFS participants), .92 (asthma participants) and .94 (healthy controls). Cronbach’s alpha for the trait items was .92 (CFS participants), .94 (asthma participants) and .93 (healthy controls).

#### Depression

The Children’s Depression Inventory (CDI; Kovacs, 1992) is a self-report scale, composed of 27 items. Each item is rated on a 3-point scale (scored 0–2). The CDI encompasses negative mood, ineffectiveness, anhedonia, low self-esteem and interpersonal problems. The recall period is the past fortnight. Higher scores indicate more depressive symptoms. The CDI is reliable and valid (Kovacs, 1992). Cronbach’s alpha was .90 (CFS participants), .85 (asthma participants) and .84 (healthy controls).

#### Catastrophising

Catastrophising about symptoms (for example, ‘I worry that I may become permanently bedridden because of my symptoms’) was assessed using the 4-item catastrophising subscale of the Cognitive and Behavioural Responses Questionnaire (Moss-Morris & Chalder, 2003; Ryan et al., 2018). Respondents are asked to respond to each item on a 5-point scale (0 = *strongly disagree*, 4 = *strongly agree*). Each item is presented as a statement (e.g., ‘My illness is awful and I feel that it overwhelms me’). Item scores are summed to generate a total subscale score. Higher scores indicate more catastrophising about symptoms. Cronbach’s alpha was .78 (CFS participants) and .78 (asthma participants). Due to the focus of this measure on symptoms, it was not administered to healthy controls.

### Procedure

#### CFS patients

Questionnaires and an invitation letter, describing the use of the data for research, were sent to all patients scheduled to attend an initial clinical assessment appointment at two specialist CFS units. Subsequently, the health care professional conducting the assessment provided the adolescent and their family with the study information sheet, sought their consent to participate and gathered the completed questionnaires. Questionnaires were re-administered when the patient next attended the unit for a first treatment appointment. Thus, we considered follow-up to be naturalistic as treatment had not commenced prior to questionnaire re-administration. Some patients were not followed up due to funding issues, or because they did not require or want treatment. Eighty (66.2%) completed the measures including the insomnia scale at their first treatment appointment, approximately 3 months later (*M* = 3.29, *SD* = 2.05, range: 0.89–13.60).

#### Asthma patients

General practitioner (GP) surgeries identified potentially eligible participants and posted them an invitation letter and research pack, which they returned to the study team by post. Seven GP surgeries sent a total of 413 letters to potential participants, and 39 of these responded to the letter, 31 of whom were eligible to participate. Eight potential participants were ineligible as they were not prescribed medication for asthma.

#### Healthy controls

Six secondary schools identified potentially eligible participant and sent them an invitation letter and a research pack. A total of 1228 letters were sent out. This resulted in 97 responses from potential participants, of whom 76 completed the questionnaires included in the current study. Where they met the eligibility criteria, the relatives of clinic staff were also invited to participate (*N* = 2).

### Ethical approval

National Health Service (NHS) research ethics committee (LREC, ref 08/H0807/107) and local research and development department approval was obtained. The local NHS clinical audit committee also approved the collection of routine outcomes.

## Data analysis plan

Data were analysed using SPSS 24.0. Complete data were available on the Insomnia Scale for 96.9% of participants. For the CFS participants, complete data were available at baseline for most participants (98.3% catastrophising scale, 96.7% CFQ, 90.9% SF-36PFS, 85.1% STAI, 81.8% CDI). At follow-up, 93.9% of those CFS participants followed up completed all the items of the SF-36PFS, and 97.6% completed all the items on the CFQ. On a completed scale, where <25% of the data for a participant were missing, the mean of the completed items was substituted in place of the missing values (CFS participants at baseline: no imputation on catastrophising scale, 2.5% CFQ, 2.5% SF-36PFS, 14.0% STAI, 14.9% CDI; at follow-up: 2.4% CFQ, 6.1% SF-36PFS). No imputation was undertaken where ⩾25% of the data were missing and these cases were omitted from the analysis.

Groups were compared on demographic variables and variables of interest using one-way analyses of variance (ANOVAs). Post hoc pair-wise comparisons with Bonferroni corrections were conducted to establish the direction of significant findings.

A paired samples *t*-test was used to establish whether sleep disturbances changed significantly over the naturalistic follow-up period before treatment commenced in the CFS group. A hierarchical linear regression, including age, anxiety, depression, fatigue, catastrophising and time between baseline and follow-up as covariates, was conducted to explore the extent to which sleep disturbance at follow-up was accounted for by sleep disturbance at baseline.

Hierarchical linear regressions, informed by theoretical assumptions based on previous studies, were used to look at predictors of change in sleep over the follow-up period. Fatigue (CFQ) and functioning (SF-36PFS) were the outcomes of interest. Time elapsed between baseline and follow-up (time interval) and age (a proxy for pubertal status) were included as covariates, as well as fatigue/functioning at baseline, catastrophising, anxiety and depression.

### Results

A total of 121 CFS participants, 27 asthma participants and 78 healthy controls took part in the study. There was no significant difference between groups on mean age. However, the CFS participants were significantly more fatigued, had more symptoms of depression (CDI) and anxiety (state and trait subscales of STAI) and had lower physical functioning than the other two groups, who did not differ significantly from each other (Table 2).

*Hypothesis 1: Do adolescents with CFS have more self-reported sleep problems than those with asthma and healthy controls?*

**Table 2.** Between-group comparison on baseline variables and insomnia scale items.

	Group			Group difference	Direction of group differences established in post hoc tests
	CFS	Asthma	Healthy controls		
	Mean (*SD*)	Mean (*SD*)	Mean (*SD*)		
Age (years)	15.01 (1.71)	14.89 (2.24)	14.58 (1.40)	*F*(2, 223) = 1.57, *p* = .210	
Fatigue (CFQ)	23.20 (5.78)	11.89 (2.71)	10.45 (3.76)	*F*(2, 222) = 182.09, *p* < .001	CFS > asthma = HCs
Physical functioning (SF-36PFS)	49.98 (25.09)	88.52 (12.70)	90.32 (17.08)	*F*(2, 214) = 95.23, *p* < .001	CFS < asthma = HCs
Depressive symptoms (CDI total)	15.72 (8.48)	7.26 (5.76)	5.64 (5.18)	*F*(2, 219) = 50.73, *p* < .001	CFS > asthma = HCs
State Anxiety (STAI-State)	45.50 (12.59)	34.78 (10.44)	34.77 (11.44)	*F*(2, 222) = 22.51, *p* < .001	CFS > asthma = HCs
Trait Anxiety (STAI-Trait)	48.04 (11.63)	39.70 (11.39)	37.47 (11.17)	*F*(2, 222) = 21.71, *p* < .001	CFS > asthma = HCs
Catastrophising Subscale (CBRQ)	8.21 (3.43)	2.37 (2.34)	N/A	*F(*1, 144) = 70.63, *p* < .001	CFS > asthma
Insomnia Scale (IS) total	22.77 (10.30)	12.04 (10.04)	8.65 (8.80)	*F*(2, 222) = 52.68, *p* < .001	CFS > asthma = HCs
IS item 1: I find it difficult to get to sleep	2.73 (1.28)	1.11 (1.16)	1.05 (1.24)	*F*(2, 222) = 49.37, *p* < .001	CFS > asthma = HCs
IS item 2: My sleep is interrupted and disturbed	2.19 (1.40)	1.00 (1.33)	0.50 (0.91)	*F*(2, 222) = 45.76, *p* < .001	CFS > asthma = HCs
IS item 3: I wake up many times during my sleep	1.90 (1.42)	0.81 (1.15)	0.56 (1.01)	*F*(2, 221) = 28.83, *p* < .001	CFS > asthma = HCs
IS item 4: I wake up early in the morning before getting enough sleep	1.36 (1.34)	1.15 (1.13)	0.77 (1.19)	*F*(2, 220) = 5.16, *p* = .006	CFS > asthma = HCs
IS item 5: I feel depressed when it is time for me to go to bed	0.85 (1.21)	0.70 (1.03)	0.44 (0.88)	*F*(2, 221) = 3.44, *p* = .034	CFS > asthma = HCs
IS item 6: Before I go to sleep I have bad thoughts	1.03 (1.25)	0.74 (1.13)	0.54 (0.96)	*F*(2, 221) = 4.51, *p* = .012	CFS > asthma = HCs
IS item 7: I feel tired when I wake up	3.45 (0.86)	1.85 (1.29)	1.69 (1.32)	*F*(2, 222) = 69.15, *p* < .001	CFS > asthma = HCs
IS item 8: I normally wake up in a bad mood	1.83 (1.39)	0.96 (1.22)	0.85 (1.17)	*F*(2, 222) = 14.95, *p* < .001	CFS > asthma = HCs
IS item 9: I get tense when I wake up	1.18 (1.41)	0.70 (1.17)	0.42 (0.81)	*F*(2, 222) = 9.67, *p* < .0001	CFS > asthma = HCs
IS item 10: My interrupted sleep annoys me	2.39 (1.50)	1.37 (1.50)	0.72 (1.19)	*F*(2, 222) = 34.45, *p* < .001	CFS > asthma = HCs
IS item 11: My interrupted sleep affects my relationships with others	1.45 (0.48)	1.44 (0.64)	0.13 (0.12)	*F*(2, 222) = 23.41, *p* < .001	CFS > asthma = HCs
IS item 12: My interrupted sleep affects my work performance	2.35 (1.40)	1.15 (0.95)	0.77 (1.08)	*F*(2, 221) = 40.46, *p* < .001	CFS > asthma = HCs

CDI: Children’s Depression Inventory; CFQ: Chalder Fatigue Questionnaire; HCs: healthy controls; IS: Insomnia Scale; SF-36PFS: Short Form 36 Physical Functioning Scale; STAI: State-Trait Anxiety Inventory; CFS: chronic fatigue syndrome; *SD*: standard deviation; CI: confidence interval; *SE*: standard error.

As predicted, participants with CFS scored significantly higher on the Insomnia Scale than healthy controls and participants with asthma (see Table 2). This was true of each of the 12 individual items on the Insomnia Scale as well as for the total score (Table 2).

*Hypothesis 2: Does baseline insomnia predict subsequent insomnia score?*

In the CFS group, 80 (66.2%) participants completed follow-up measures including the Insomnia Scale. Those who completed the Insomnia Scale at follow-up did not differ significantly from those who were not followed up/did not complete the insomnia scale at follow-up (Table 3). In those CFS participants who were followed up, there was a non-significant trend for self-reported sleep difficulties on the Insomnia Scale to decrease from baseline (*M* = 23.15, *SD* = 10.48) to pre-treatment follow-up (*M* = 21.60, *SD* = 11.16, *t* = 1.97, *p* = .053). A hierarchical linear regression found that 60.5% of the variance in insomnia scale score at follow-up was predicted by the combination of depression, trait anxiety and catastrophising at baseline. More depressive symptoms, higher trait anxiety and more catastrophising about symptoms predicted greater insomnia. Fatigue and state anxiety at baseline, age and time interval did not significantly contribute to the variance explained. A further 12.8% of the variance was predicted by baseline insomnia scale score (Table 4).

*Hypothesis 3: Do elevated sleep disturbances at baseline predict fatigue and functioning at follow-up?*

**Table 3.** Comparison of means between CFS participants with and without follow-up sleep data.

	CFS mean (*SD*) – with follow-up	CFS mean (*SD*) – not followed up	Significance tests – *t* (df)	Significance level (*p*)	Mean difference (95% CI)	*SE* of mean difference
Age	14.96 (1.76)	15.10 (1.61)	0.41 (119)	.682	0.14 (–0.52 to 0.79)	0.33
CFQ	23.18 (5.84)	23.23 (5.72)	0.04 (118)	.970	0.04 (–2.18 to 2.27)	1.12
SF-36PFS	51.35 (24.79)	47.13 (25.82)	–0.84 (111)	.404	–4.22 (–14.20 to 5.76)	5.04
CDI Total	16.42 (8.83)	14.33 (7.65)	–1.26 (115)	.211	–2.09 (–5.37 to 1.20)	1.66
STAI-S	46.84 (11.90)	42.83 (13.62)	–1.66 (118)	.100	–4.01 (–8.80 to 0.78)	2.42
STAI-T	48.79 (11.81)	46.52 (11.26)	–1.01 (118)	.316	–2.27 (–6.73 to 2.19)	2.25
CBRQ Catastrophising Subscale	8.08 (3.70)	8.48 (2.84)	0.60 (117)	.551	0.40 (–0.92 to 1.72)	0.67
Insomnia Scale	22.93 (10.37)	22.47 (10.27)	–0.23 (118)	.818	–0.46 (–4.40 to 3.48)	1.99

Two-tailed tests.

CBRQ: Cognitive and Behavioural Responses Questionnaire; CDI: Children’s Depression Inventory; CFQ: Chalder Fatigue Questionnaire; SF-36PFS: Short Form 36 Physical Functioning Subscale; STAI: State-Trait Anxiety Inventory; CFS: chronic fatigue syndrome; *SD*: standard deviation; CI: confidence interval; *SE*: standard error.

**Table 4.** Hierarchical linear model of predictors of outcome at time 2.

	Unstandardised *B*	*SE B*	Standardised Beta	*T*	*p*
Outcome: Time 2 Insomnia					
Step 1					
Constant	13.34	9.41		1.42	.162
Time between T1 and T2	0.34	0.45	.07	0.76	.453
Fatigue (CFQ) T1	0.29	0.18	.15	1.64	.107
Depression (CDI)	0.95	0.17	.78	5.76	<.001
State anxiety (STAI-S)	0.22	0.15	.23	1.46	.150
Age (years)	–0.66	0.55	–.11	–1.20	.234
Trait anxiety (STAI-T)	–0.44	0.19	–.47	–2.33	.023
Catastrophising (CBRQ)	0.77	0.33	.26	2.31	.025
*r*^2^ = .605, *p* < .001					
Step 2					
Constant	12.12	7.71		1.57	.122
Time between T1 and T2	0.40	0.37	.08	1.07	.291
Fatigue (CFQ) T1	0.20	0.15	.11	1.37	.178
Depression (CDI)	0.52	0.16	.43	3.27	.002
State anxiety (STAI-S)	0.22	0.12	.23	1.80	.078
Age	–0.82	0.45	–.13	–1.83	.073
Trait anxiety (STAI-T)	–0.44	0.16	–.46	–2.80	.007
Catastophising (CBRQ)	0.68	0.27	.23	2.48	.017
T1 Insomnia Scale	0.57	0.11	.53	5.20	<.001
*r*^2^ = .753, *r*^2^ change = .128, *p* < .001					
Outcome: Time 2 Fatigue					
Step 1					
Constant	17.07	6.73		2.54	.014
Time between T1 and T2	–0.56	0.33	–.18	–1.69	.097
Age	–0.36	0.40	–.10	–0.90	.373
Trait anxiety (STAI-T)	–0.05	0.13	–.09	–0.39	.698
State anxiety (STAI-S)	–0.04	0.11	–.07	–0.38	.708
Depression (CDI)	0.37	0.12	.51	3.10	.003
T1 fatigue (CFQ)	0.64	0.12	.57	5.45	<.001
*r*^2^ = .416, *p* < .001					
Step 2					
Constant	17.01	6.67		2.55	.014
Time between T1 and T2	–0.57	0.33	–.18	–1.72	.091
Age	–0.30	0.39	–.08	–0.77	.447
Trait anxiety (STAI-T)	–0.05	0.13	–.08	–0.35	.728
State anxiety (STAI-S)	–0.04	0.11	–.07	–0.39	.699
Depression (CDI)	0.47	0.14	.65	3.41	.001
T1 fatigue (CFQ)	0.49	0.13	.44	3.87	<.001
T1 Insomnia Scale	–0.13	0.10	–.21	–1.41	.165
*r*^2^ = .437, *r*^2^ change = .02, *p* = .165					
Outcome: Time 2 Physical Functioning (SF-36PFS)					
Step 1					
Constant	8.92	20.17		0.44	.660
Time between T1 and T2	2.45	1.00	.20	2.46	.017
Age	0.18	1.19	.01	0.16	.877
T1 SF-36PFS	0.84	0.09	.79	9.85	<.001
Trait anxiety (STAI-T)	–0.40	0.39	–.17	–1.01	.315
State anxiety (STAI-S)	0.33	0.33	.14	1.00	.323
Depression (CDI)	–0.32	0.37	–.11	–0.87	.391
*r*^2^ = .673, *p* < .001					
Step 2					
Constant	9.00	20.08		0.45	.656
Time between T1 and T2	2.44	0.99	.20	2.46	.017
Age	0.34	1.19	.02	0.28	.778
T1 SF-36PFS	0.85	0.09	.79	9.94	<.001
Trait anxiety (STAI-T)	–0.38	0.39	–.16	–1.00	.334
State anxiety (STAI-S)	0.33	0.33	.14	1.01	.318
Depression (CDI)	–0.05	0.43	–.02	–0.12	.903
T1 Insomnia Scale	–0.35	0.29	–.14	–1.21	.231
*r*^2^ = .682, *r*^2^ change = .009, *p* = .231					

CBRQ: Cognitive and Behavioural Responses Questionnaire; CDI: Children’s Depression Inventory; CFQ: Chalder Fatigue Questionnaire, SF-36PFS: Short Form 36 Physical Function Subscale; STAI: State-Trait Anxiety Inventory; T1: Time 1 (baseline); T2: Time 2 (follow-up).

A hierarchical linear regression in which fatigue at time 2 was the outcome of interest found that the combination of a smaller time interval, younger age, higher baseline fatigue and higher baseline depression accounted for 41.6% of the variance in fatigue at time 2. Baseline anxiety explained little of the variance. Baseline insomnia score explained a further 2.1% of the variance (see Table 4). For physical functioning at time 2, a larger time interval and better baseline physical functioning explained 67.3% of the variance. Only a further 0.9% was explained by baseline insomnia scale score (see Table 4).

## Discussion

**Figure 1.**
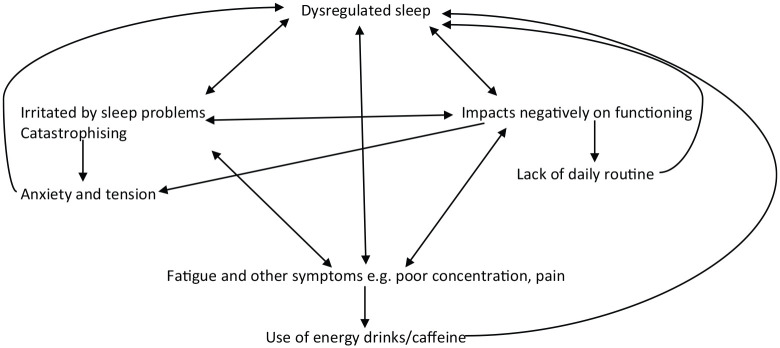
Proposed vicious cycle of sleep.

We found that adolescents with CFS reported increased sleep disturbances in all aspects of sleep compared to both healthy adolescents and illness controls (adolescents with asthma). Specifically, adolescents with CFS had a higher total score on the Insomnia Scale, with difficulties reported on the items assessing getting to sleep, sleep disturbance and night-time awakenings, as well as more tiredness on waking. Adolescents with CFS also reported that their sleep problems had greater consequences, annoying them and affecting their schoolwork performance, thus creating a vicious cycle (see Figure 1). Other factors such as strategies that adolescents may use in an attempt to overcome fatigue, such as consuming caffeine or energy drinks (Owens, 2014), may further contribute to this, although this has not been explicitly investigated in adolescents with CFS. We found that sleep disturbances tended to decrease over a naturalistic follow-up period in the CFS participants. Nevertheless, sleep disturbance at baseline predicted a significant proportion of the variance in sleep problems at follow-up, even when other known covariates of sleep were accounted for, including mood, anxiety, catastrophising, fatigue, age (as a proxy for pubertal status) and the time elapsed between baseline and follow-up. However, sleep disturbances at baseline did not account for much of the variance in fatigue or functioning at follow-up.

Several studies have found that adolescents with CFS report greater sleep disturbances than healthy controls (Josev et al., 2017; Pedersen et al., 2017; Snodgrass et al., 2015). Our findings have further confirmed these existing conclusions. Furthermore, the inclusion of an illness control group as well as a healthy control comparison has allowed us to build on existing studies, as the CFS group differed from another chronic illness condition; hence, it appears that sleep disturbances may not simply be accounted for by a chronic illness.

Across studies using a range of self-report and objective measures, the presence of sleep disturbances in adolescents with CFS is a consistent finding. Sleep disturbances are part of the diagnostic criteria for CFS (NICE, 2007); the findings of the current study and those of Josev et al. (2017) and Pedersen et al. (2017) have particularly illustrated the breadth of those difficulties across various facets of sleep, including difficulties falling asleep, staying asleep and feeling unrefreshed on waking up. In the short term, sleep problems are known to impact on well-being, school performance and risk-taking behaviours in the general population (Medic et al., 2017). Long-term, sleep disruption impacts negatively on physical health and on health-related quality of life (Medic et al., 2017), including in adolescents with chronic pain (Palermo et al., 2012). This highlights the importance of further investigating sleep problems in CFS and potentially in tackling sleep as a treatment target.

Anxiety and depression have been linked to sleep disturbance in adolescents generally (Alfano et al., 2007, 2009; Lovato & Gradisar, 2014; Lovato et al., 2017) and in adolescents with chronic pain specifically (Palermo & Kiska, 2005). In our study, it was the combination of depression, trait anxiety and catastrophising that explained most of the variance in sleep problems subsequently, with state anxiety not contributing significantly to the variance explained. There is considerable research demonstrating that sleep difficulties contribute to subsequent anxiety (Gregory et al., 2005; Hertenstein et al., 2019) and depression (Baglioni et al., 2011; Lovato & Gradisar, 2014), but there has been less evidence for anxiety and/or depression predicting subsequent sleep difficulties, although a bidirectional relationship is generally acknowledged (Owens, 2014). It may be that experiencing emotional distress in addition to CFS compounds the disruption to sleep over time. It may also be that the profound impact of CFS on functioning can lead to low mood (Taylor et al., 2017) and that sleep disturbances arise as part of the mood disorder. Treating emotional distress, which is common in adolescents with CFS (Loades et al., 2018), may therefore be a particular priority.

Previous studies have not examined the predictive value of sleep problems over time in adolescents with CFS. Our finding that sleep problems persist over a naturalistic follow-up period is important and further indicates that these difficulties are a key treatment target, as is recommended by NICE (2007). Sleep problems are often tackled at the outset of treatment (Baos et al., 2018; Brigden et al., 2016; Lloyd et al., 2012; Nijhof et al., 2011). It is interesting that in the current study, self-reported sleep problems did not particularly predict variance in fatigue or functioning. Instead, depression appeared to particularly contribute to the variance in fatigue (Loades, Rimes, Ali, & Chalder, 2019).

The measure of sleep we used did not allow us to investigate some potentially important aspects of sleep habits, such as having a nap during the day, and the impact of daytime activities (or lack of due to fatigue) on night-time sleepiness. It may be that the lack of structured activities during the day in CFS affects the ‘body clock’, and vice versa, that sleep problems affect the ability to do daytime activities. If this were found to be the case, regulating sleep could influence outcomes not assessed in this study such as school attainment and social functioning. However, it is more difficult to regulate sleep in the absence of a structured routine, which is provided by school attendance and other activities. Introducing a basic structure to the day, within the limits of fatigue, as is done in activity management interventions (Brigden et al., 2017), could be a useful step in regulating sleep. Future research could explore these aspects further by the additions of measures such as the Adolescent Sleep Hygiene Scale (Storfer-Isser et al., 2013), as well as the School and Social Adjustment Scale (Loades et al., 2020).

Beliefs about sleep are associated with sleep problems in children (Gregory et al., 2009) and young adults (Yang et al., 2011). Furthermore, cognitive processes such as catastrophising have also been linked to sleep disturbances in adolescents (Hiller et al., 2014). Our current findings indicate that adolescents with CFS who catastrophise more about their symptoms were more likely to endorse sleep difficulties subsequently. Future research to further explore the impact of such cognitive processes on sleep in CFS could help to refine treatments for sleep in this population by furthering the understanding of how sleep problems are maintained.

## Strengths and limitations

Recruitment was via specialist CFS units, but this does mean that the findings are most relevant to those presenting to specialist services. There was some ethnic variation in all groups, as recruitment took place in a metropolitan area in the United Kingdom. However, generalisations to other settings should be made with caution. Although asthma has several similarities to CFS, as a chronic illness with a relatively unpredictable and fluctuating course, quality of life may not be so severely affected by asthma as by CFS. Future studies could benefit from using other more debilitating health conditions as a control group. It is possible that the asthma control group we recruited was a biased comparison sample due to the eligibility criteria (i.e., need to be prescribed medication) and recruitment setting (primary care) and method (opt in). Controls were not specifically matched by age or gender.

Sleep quality was assessed using self-report only, which may not necessarily correlate with objective measures (Josev et al., 2017). Future studies would ideally assess sleep both objectively and subjectively. We also did not measure body mass index, substance use including caffeine intake, physical activity levels or use of medications, which may impact on sleep and it would be important for this to be addressed in future studies. We used age as a proxy for pubertal status, but other methods as hormonal levels and Tanner staging may be more accurate.

Only a subsample of the CFS participants attended follow-up, and the follow-up period varied, although we did control for this in the analysis. Furthermore, although treatment had not started at the point of follow-up, an assessment had been completed and treatment had been offered, which could in itself act as an intervention. We selected the best measures available at the time of the study, but the measures had not been specifically validated for use in adolescents with CFS. Although CFS participants were clinically assessed and had also seen a Paediatrician prior to referral who had confirmed that the main presenting complaint was fatigue, we cannot rule out the possibility of a primary sleep disorder in any of the participant groups as we did not formally assess this.

We opted to use imputation for missing data. This assumes that the missing values were missing at random; if this were not the case, this strategy may have skewed the results. However, when the analyses were re-run on the raw data, the direction and significance of the findings remained the same.

## Clinical and research implications

Our findings highlight the importance of assessing and attending to sleep problems in adolescents with CFS. By remediating sleep problems, the vicious cycle (Figure 1) could be addressed. Sleep can be improved by stabilising the wake up time and limiting the number of hours spent in bed to those required (age-dependent), which addresses excessive sleeping, enabling time for sleep pressure to build during waking hours and thus contributing to better night-time sleep (Brigden et al., 2017). In addition, introducing a consistent bedtime routine and avoiding the use of bright screens prior to bedtime can be helpful. Time spent lying awake in bed (for example, playing games, watching videos or worrying) would be minimised to maintain the association between bed and sleep. Treatments such as Cognitive Behaviour Therapy for insomnia (CBT-I), which has been shown to remediate sleep problems in adolescents in the general population (de Bruin et al., 2015), may hold promise for use in adolescents with CFS. Many of the principles used in CBT-I are the same as those used to manage sleep in CFS (NICE, 2007). Few existing treatment studies in adolescents with CFS have utilised measures of sleep as outcome measures; further research to examine the impact of different forms of treatment on sleep, both subjectively reported sleep and objective measures of sleep, would enable conclusions to be drawn about what may be most helpful in addressing sleep difficulties in this population.

## Conclusion

Adolescents with CFS appear to experience more self-reported sleep problems than illness controls or healthy controls, and these sleep problems appear to persist over time. As sleep problems are known to be associated with a range of unfavourable outcomes, it is important that sleep problems in CFS are tackled as part of treatment. It is also important for future studies, including treatment trials in adolescents with CFS, to include measures of sleep as an outcome.
